# Reevaluating the presidential runoff rule: Does a provision promote the protection of human rights?

**DOI:** 10.1371/journal.pone.0217650

**Published:** 2019-05-31

**Authors:** Joshua Holzer

**Affiliations:** Westminster College, Fulton, MO, United States of America; Georgia State University, UNITED STATES

## Abstract

In recent years, an increasing number of democracies have adopted a runoff rule to elect their president. Some have argued, however, that the benefits of such a rule are dubious at best. In this article, I seek to counter this claim, as I posit that a runoff rule *promotes* the protection of human rights by reducing outcomes that are *negatively* associated with high government respect for human rights. Using ordered logistic regression and an analysis of predicted probabilities, I find that democratic presidential elections held using a runoff rule produce presidents that are *less* likely to be associated with *lower* government respect for human rights, and *more* likely to be associated with *greater* government respect for human rights. I conclude by suggesting that politicians should consider embracing a presidential runoff rule, as its adoption could be a relatively easy way to reduce repression.

## Introduction

Today, “[t]he runoff electoral system is the single most used electoral system for presidential elections” ([1]: 1248). This system is “especially prevalent among those regimes that previously had presidents elected by plurality, but then lapsed into a period of authoritarianism” ([2]: 323–324). The thought seems to be that perhaps a runoff rule would give citizens *two* chances—instead of one—to avoid accidentally electing a leader with authoritarian aspirations. Recent research, however, argues that “[t]he need for runoff elections is dubious” ([3]: 129). In this article, I seek to counter this claim, as I posit that a runoff rule *promotes* the protection of human rights by reducing outcomes that are *negatively* associated with high government respect for human rights. Using ordered logistic regression and an analysis of predicted probabilities, I find that democratic presidential elections held using a runoff rule produce presidents that are *less* likely to be associated with *lower* government respect for human rights, and *more* likely to be associated with *greater* government respect for human rights. Bouton ([1]: 1249) notes that “despite the relative ubiquity of runoff systems, our understanding of their properties…is limited.” With this article, I add to the “scant empirical literature on…runoff elections” ([4]: 284), as my results suggest that democracies with such a provision would be wise to retain it, while those that have not yet adopted a runoff rule should consider doing so.

## Theoretical argument

In this article, I argue that it is not whether an election advances to runoff round that is important for human rights, but rather whether the election *could* have advanced to a runoff round. In other words, I argue that it is not the runoff *round* that promotes the protection of human rights, but rather the presence of a runoff *rule*. To explain this, I have developed a theoretical framework of six premises, which together build up to my hypothesis. The first three premises relate to recent papers of mine that find government respect for human rights in presidential democracies to be affected by the president’s ideology, the composition of the president’s cabinet, and whether or not the president was elected by a majority [5–7]. My first premise is that president’s who are ideological *distant* from the median voter are *less* likely to be associated with high government respect for human rights. My second premise is that cabinets comprised of a high percentage of individuals in the *same* party as the president are *less* likely to be associated with high government respect for human rights. Finally, my third premise is that presidents elected *without* a majority are *less* likely to be associated with high government respect for human rights.

In addition to the three premises above, I advance three additional premises regarding outcomes that result from a runoff rule. For instance, Hotelling ([8]: 54) suggests that in a two-candidate race using plurality rule, “each [candidate’s] party strives to make its platform as much like the other as possible.” Boulding ([9]: 484) names this phenomenon “[t]he Principal of Minimum Differentiation.” In a later study, Osborne ([10]: 284) adds that “when there are more than two (potential) candidates, then the basic incentive [for minimum differentiation] inherent in the Hotelling model is significantly diluted.” Instead, the incentive is for candidates to disperse along the ideological spectrum. Haan and Volkerink ([11]: 161), however, argue that “a runoff system restores the principle of minimum differentiation.” Essentially, when elections are held using a runoff rule, the incentive is for politicians to strive and make their platforms as minimally different as possible because if a politician were to adopt any position other than that of the median voter, “[i]n the runoff round…he [or she] will be beaten by one of the candidates that did choose the median voter’s position” ([11]: 160). In other words, politicians that are ideologically “closer to the median voter’s ideal point are most likely to win” ([12]: 459). This leads me to my fourth premise: a presidential runoff rule is *unlikely* to lead to a president that is ideological *distant* from the median voter. Note that per Hotelling [8]—and later Downs [13]—a two-candidate race using plurality rule is *also* unlikely to lead to a president that is ideological distant from the median voter; however, in practice two-candidate races using plurality rule are *exceedingly* rare in modern presidential races. If even one additional candidate enters a two-candidate race using plurality rule (as is almost always the case), the incentives change; *defecting* from the median voter’s position becomes optimal, as defecting is most likely to maximize vote share [14].

Next, although the conventional view has attributed cabinet composition in presidential systems to institutional characteristics, such as electoral rules, formal powers of the president, and party characteristics [15–17], Freudenreich ([18]: 80) “argues that the partisan composition of cabinets is largely predetermined by the bargaining and the competition before and during presidential elections.” Pérez-Liñán ([3]: 132) adds that a runoff rule encourages “the formation of inclusive electoral alliances before the second round—alliances that may easily become the basis for coalition governments.” As Linz ([19]: 57) explains,“run-off provisions…set up a confrontation between the two major candidates,” and as a result, “broad coalitions are likely to be formed…in preelection maneuvering.” This leads me to my fifth premise: a presidential runoff rule is *unlikely* to lead to a cabinet with a *high* percentage of individuals in the same party as the president.

Finally, in states that have a runoff rule, typically if no one candidate receives more than 50% of the vote in the first round, then the top two finishers advance to a runoff election. Notably, however, there are a few exceptions to this. For instance, in Argentina, a candidate with 45% of the vote can avoid a runoff, or they can also avoid a runoff by netting 40% of the vote and having at least a 10% lead over the second-place candidate. In Costa Rica, a candidate simply needs 40% of the vote to avoid a runoff. Finally, up until recently a candidate in Nicaragua with 40% of the vote could avoid a runoff, or they could also avoid a runoff by netting 35% of the vote and having at least a 5% lead over the second-place candidate. The point is, while it *is* possible that an election held using a runoff rule *could* produce a president elected with less than 50% of the vote, in practice, this is quite rare. More often, a presidential election held using a runoff rule produces a president that obtains at least 50% of the vote. This leads me to my sixth premise: a presidential runoff rule is *unlikely* to produce a president elected *without* a majority.

In Table 1, I review my theoretical argument. In essence, I argue that the presence of a runoff rule *reduces* the following: the distance between the president and the median voter, the percent of the cabinet in the same party as the president, and finally the likelihood that the president is elected without a majority. Furthermore, note that each of these three outcomes are likely to be *negatively* associated with high government respect for human rights. If the presence of a runoff rule *reduces* outcomes that in turn are *negatively* associated with high government respect for human rights, then a runoff rule should be *positively* associated with high government respect for human rights. This leads me to my hypothesis:

In comparison to when the president is elected using plurality rule, when the president is elected using a runoff rule, that state is *more* likely to be associated with high government respect for human rights.

**Table 1:** Explaining the relationship between presidents elected using a runoff rule and government respect for human rights.

**Premises**				
President is elected using a runoff rule	→ reduces →	President’s distance from the median voter	→ negative →	Government respect for human rights
President is elected using a runoff rule	→ reduces →	Percent of the cabinet in the same party as the president	→ negative →	Government respect for human rights
President is elected using a runoff rule	→ reduces →	President is elected without a majority	→ negative →	Government respect for human rights
**Hypothesis**				
President is elected using a runoff rule	→→→	→→ positive →→	→→→	Government respect for human rights

## Methods

### Variables

In order to test my hypothesis, I have constructed the following independent variable: *president is elected using a runoff rule*. To construct this variable, I consulted the Democratic Electoral Systems Around the World dataset [20]. For each country-year, I looked up the most recent democratic election which had brought the current president into power. If, during that country-year, a runoff rule had been in place, my *president is elected using a runoff rule* variable was coded as ‘1’. For each country-year where the previous election did *not* have a runoff provision (i.e., it was held using plurality rule), this variable was coded as ‘0’.

For the dependent variable of my hypothesis, I utilize the Cingranelli-Richards (CIRI) Physical Integrity Rights Index [21]. This is an additive index created from four individual indicators (torture, extrajudicial killing, political imprisonment, and disappearance), which ranges from ‘0’ (which indicated no respect for any of these four rights) to ‘8’ (which indicates full respect for these four rights). In this article, I refer to this index as the *CIRI scale*. Note that the *CIRI scale* is only available up until 2011, which provides a temporal limitation of this study.

To test my hypothesis, I include a series of control variables, all of which are standard within the human rights literature. For instance, I include Doyle and Elgie’s [22] measure of *presidential power* to account for the differing levels of executive authority that exist across different presidential systems. Second, a measure of *civil conflict*, which is drawn from the Uppsala Conflict Data Program [23]. This variable is coded as ‘0’ for each country-year with less than 25 battle-related deaths, ‘1’ for each country-year where there were between 25 and 999 battle-related deaths, and finally ‘2’ for each country-year where there were more than 999 battle-related deaths. Next, (logged) measures of population size and gross domestic product (GDP) per capita, both of which are from the World Bank [24]. Finally, since “[p]atterns of abuse tend to persist over time,” I follow others in “including a lagged dependent variable” ([25]: 352). I refer to this variable as: *previous year’s score on the CIRI scale*.

In order to test the premises that provide the basis of my theoretical argument, I use three variables that I previously constructed for my earlier works [5, 6]). For the first variable—which is the *president’s distance from the median voter*—values closer to ‘0’ indicate that the president and the median voter are ideologically similar, while values closer to ‘1’ suggest that the president is ideological *distant* from the median voter. In order to construct this variable, I followed Kim and Fording [26, 27], who outlined a method to estimate the position of the median voter. Using data from the Manifesto Project [28, 29], I then measured the ideological distance between the position of the median voter and the position of the candidate that won the election (see [5] for further details).

For the second variable I previously constructed—which is the *percent of the cabinet in the same party as the president*—a value of ‘0’ indicates that 0% of the cabinet is in the same party as the president, while a ‘1’ indicates that 100% of the cabinet is in the same party as the president. In order to construct this variable, I first used the Chiefs of State and Cabinet Members of Foreign Governments directory [30] to determined who was in each cabinet for each country-year of available data. I then consulted the following sources to identify the political party affiliation of each cabinet-member: Banks, Muller, and Overstreet [31–34]; Banks, Muller, Overstreet, and Isaccoff [35–37]; Muller, Isacoff, and Lansford [38]; and Bértoa [39]. Finally, for each country-year, I simply calculated the percent of each cabinet that was comprised of cabinet members in the same party as the president.

Regarding the third variable I previously constructed—*president is elected without a majority*—for each country-year where the president received *less* than 50% of the vote (in the most recent election), that country-year was coded as ‘1’, while all country-years where the president had been elected with *more* than 50% of the vote were coded as ‘0’. This data was gathered from previously mentioned sources sources [31–38].

### Analyses

Before testing my hypothesis, I first test the premises that underpin my theoretical argument. Recall from Table 1 that I assume that presidents elected using a runoff rule are *unlikely* to be ideological *distant* from the median voter. Consistent with this premise, Table 2 reports that there is a statistically significant difference between the mean *president’s distance from the median voter* score for presidents that were elected using a runoff rule versus the mean *president’s distance from the median voter* score of presidents that were elected using plurality rule. As you can see, presidents elected using a runoff rule appear to be ideologically less distant from the median voter (mean of 0.278) versus presidents elected using plurality rule (mean of 0.449). With that said, note that due to data limitations (which I detail below), this particular sample only included two countries that used plurality rule.

**Table 2:** Testing the relationship between the president being elected using a runoff rule and the president’s distance from the median voter using a two-sample *t*-test, 1990-2011.

President is elected using	President’s distance from the median voter		Total	
	Mean	95% Confidence Interval	Observations	Countries
plurality rule	0.449	[0.329, 0.570]	25	2
a runoff rule	0.278	[0.233, 0.323]	139	12
Combined	0.304	[0.261, 0.347]	164	14
*t*-statistic = 2.914***				

* *p* < 0.10,

** *p* < 0.05,

*** *p* < 0.01.

Per Table 1, note that I also assume that presidents elected using a runoff rule are *unlikely* to have a cabinet comprised of a *high* percentage of individuals in their same party. Consistent with this premise, Table 3 reports that there is a statistically significant difference between the mean *percent of the cabinet in the same party as the president* score for presidents that were elected using a runoff rule versus the mean *percent of the cabinet in the same party as the president* score of presidents that were elected using plurality rule. As you can see, presidents elected using a runoff rule appear to have less of their cabinet comprised of copartisans (mean of 0.401) versus presidents that were elected using plurality rule (mean of 0.613).

**Table 3:** Testing the relationship between the president being elected using a runoff rule and the percent of the cabinet in the same party as the president using a two-sample *t*-test, 2001-2011.

President is elected using	Percent of the cabinet in the same party as the president		Total	
	Mean	95% Confidence Interval	Observations	Countries
plurality rule	0.613	[0.551, 0.674]	54	7
a runoff rule	0.401	[0.355, 0.447]	187	28
Combined	0.448	[0.409, 0.488]	241	35
*t*-statistic = 5.515***				

* *p* < 0.10,

** *p* < 0.05,

*** *p* < 0.01.

Also per Table 1, note that I assume that presidents elected using a runoff rule are *unlikely* to have been elected *without* a majority. As you can see in Table 4, there is a statistically significant nonrandom association between these two variables. Looking more closely at this table, you can see that when the president had been elected using plurality rule, roughly 53% of the time that president had also been elected without a majority. In contrast, when the president had been elected using a runoff rule, only about 6% of the time had that president also been elected without a majority. While this result may look strange, recall that in some states with a runoff rule (such as Argentina, Costa Rica, and Nicaragua, which is mentioned above), the threshold for ‘triggering’ a runoff round is below the typically-standard threshold of 50%. For such states, it is possible that a candidate could win the election *without* a majority (and *without* triggering a runoff round), although this is quite rare (which is why Table 4 reports that this is the case for only 6% of the country-years in the time period examined). In most states with a runoff rule, however, if any candidate does not surpass 50% in the first round, a second round is required, and in that second round, one of the two candidates is *guaranteed* to obtain at least 50% of the vote (as was the case for 94% of the country-years in the time period examined).

**Table 4:** Testing the relationship between the president being elected using a runoff rule and the president being elected without a majority using Fisher’s exact test, 1990-2011.

President is elected using	President is elected		Total	
	with a majority	without a majority	Observations	Countries†
plurality rule	94 [47%]	106 [53%]	200	13
a runoff rule	589 [94%]	39 [6%]	628	42
Combined	683 [82%]	145 [18%]	828	52
*p* < 0.001				

^†^Note that the total number of countries do not add up to 52 because three countries are in both categories (i.e. they adopted a runoff rule during time period examined).

Tables 2, 3 and 4 all appear to confirm the three premises that make up the left-side of Table 1. With Models 1, 2, and 3 in Table 5, I test the final three premises that make up the right-side of Table 1 using the same variables that were used in Tables 2, 3 and 4. Recall that these final three premises seek to replicate the findings of two recent papers [5, 6]; the results that Models 1, 2, and 3 report are consistent with the results reported in these papers.

**Table 5:** Ordered logit estimates of CIRI scores in presidential democracies.

	Model 1	Model 2	Model 3	Model 4
President’s distance from the median voter	-1.202*** (0.370)			
Percent of the cabinet in the same party as the president		-0.859** (0.428)		
President is elected without a majority			-0.331** (0.159)	
President is elected using a runoff rule				0.435** (0.209)
Presidential power	-2.363*** (0.894)	-0.244 (0.785)	-1.765*** (0.563)	-1.813*** (0.587)
Civil conflict	-5.744*** (0.669)	-1.763*** (0.499)	-1.462*** (0.247)	-1.419*** (0.253)
(Logged) population size	-0.512*** (0.161)	-0.322*** (0.124)	-0.345*** (0.081)	-0.352*** (0.084)
(Logged) GDP per capita	0.973* (0.525)	0.370** (0.161)	0.174* (0.096)	0.161* (0.095)
Previous year’s score on the CIRI scale	1.145*** (0.278)	1.649*** (0.162)	1.136*** (0.102)	1.112*** (0.099)
Countries	14	35	52	52
Observations	164	241	828	828

* *p* < 0.10,

** *p* < 0.05,

*** *p* < 0.01.

Figures in parentheses are robust standard errors clustered by country. Note that higher values of the dependent variable indicate greater government respect for human rights.

Note that in Table 5, each of the models discussed up to this point (i.e. Models 1-3) all report a substantially different number of observations and countries used. A keen reader may notice that the number of observations reported (and the number of countries used) in Model 1 is the same number of observations reported (and total number of countries used) in Table 2. Similarly, the number of observations reported (and the number of countries used) in Model 2 is the same number of observations reported (and total number of countries used) in Table 3. Finally, the number of observations reported (and the number of countries used) in Model 3 (and Model 4 for that matter) is the same number of observations reported (and total number of countries used) in Table 4. A keen reader may also notice that the time period used for Tables 2, 3, and 4 also vary: the titles of Tables 2 and 4 indicate a time period of 1990-2011, while Table 3 indicates a time period of 2001-2011.

The reason for these differences in observations reported, countries used, and time periods examined ultimately lies with data limitations. For instance, data from the Manifesto Project [28, 29], which is used to construct my *president’s distance from the median voter* variable is not available for any countries outside of Europe or South America (and, in fact, coverage is not even complete for all countries *within* Europe and South America). Data from the Chiefs of State and Cabinet Members of Foreign Governments directory [30], which is used to construct my *percent of the cabinet in the same party as the president* variable is not available prior to 2001, which imposes a narrower temporal bound for certain tests. Furthermore, coverage is not entirely complete for the sources used to calculate my *percent of the cabinet in the same party as the president* variable (see [31–39]).

It is important to keep in mind that the ultimate goal of this article is to empirically test my *hypothesis*, which again, is that in comparison to when the president is elected using plurality rule, when the president is elected using a runoff rule, that state is *more* likely to be associated with high government respect for human rights. The purpose of the six premises discussed above and illustrated in Table 1 is to provide a theoretical explanation as to *why* presidents elected using a runoff rule are more likely to be associated with high government respect for human rights. These premises are meant to use established literature to help explain causality. Although the data may be imperfect (given the just discussed differences in observations reported, countries used, and time periods examined), the purpose of Tables 2, 3 and 4—as well as Models 1, 2, and 3 in Table 5—is to demonstrate that the causal arrows illustrated in Table 1 can be empirically supported using *existing* data.

If (per Table 1) the presence of a runoff rule *reduces* outcomes that in turn are *negatively* associated with high government respect for human rights, then—per my hypothesis—a runoff rule should be *positively* associated with high government respect for human rights. The results reported by Model 4 (in Table 5) support this idea. In order to better illustrate the *substantive* effects of this model, I have outlined three scenarios in Table 6, which I have used to generate the predicted probabilities reported in Table 7. Note that the scenario parameters reported in Table 6, as well as the process I used to generate the results reported in Table 7 are detailed in one of my aforementioned papers (see [5]).

**Table 6:** Summary statistics of scenarios used in predicted probabilities analysis.

	‘Best’ scenario	‘Average’ scenario	‘Worst’ scenario
Presidential power^a^	0.116	0.326	0.650
Civil conflict^b^	0	0	1
Population size	943,263	19,234,190	195,212,643
GDP per capita	$37,500	$16,728	$5,940
Government respect for human rights in the previous year^c^	8	5	2

^a^Scores range from 0 to 1; higher values indicate more powerful presidents.

^b^Scores range from 0 to 2; higher values indicate more civil conflict.

^c^Scores range from 0 to 8; higher values indicate greater government respect for human rights.

**Table 7:** The percentage change in predicted probabilities of CIRI scores when the president is elected using a runoff rule versus plurality rule.

**‘Best’ scenario**					
	CIRI scale (change versus the previous year)				
	4 (-4)	5 (-3)	6 (-2)	7 (-1)	8 (no change)
Elected using plurality rule	0.003 [0.001, 0.006]	0.015 [0.006, 0.028]	0.060 [0.028, 0.109]	0.413 [0.284, 0.535]	0.509 [0.346, 0.671]
Elected using a runoff rule	0.002 [0.001, 0.004]	0.009 [0.005, 0.017]	0.039 [0.021, 0.066]	0.335 [0.232, 0.445]	0.614 [0.484, 0.733]
Difference (plurality→runoff)	-0.001 [-0.003, -0.000]	-0.005 [-0.014, -0.000]	-0.020 [-0.051, -0.001]	-0.078 [-0.149, -0.005]	0.105 [0.006, 0.205]
Percentage change	-35.7%	-35.9%	-34.1%	-18.9%	20.6%
**‘Average’ scenario**					
	CIRI scale (change versus the previous year)				
	3 (-2)	4 (-1)	5 (no change)	6 (+1)	7 (+2)
Elected using plurality rule	0.050 [0.028, 0.081]	0.233 [0.164, 0.310]	0.392 [0.309, 0.469]	0.216 [0.155, 0.282]	0.080 [0.045, 0.129]
Elected using a runoff rule	0.033 [0.021, 0.049]	0.174 [0.119, 0.238]	0.378 [0.297, 0.456]	0.273 [0.217, 0.331]	0.115 [0.077, 0.164]
Difference (plurality→runoff)	-0.017 [-0.039, -0.001]	-0.059 [-0.115, -0.003]	-0.013 [-0.042, 0.010]	0.057 [0.003, 0.113]	0.036 [0.002, 0.069]
Percentage change	-33.9%	-25.3%	not significant	26.4%	44.7%
**‘Worst’ scenario**					
	CIRI scale (change versus the previous year)				
	0 (-2)	1 (-1)	2 (no change)	3 (+1)	4 (+2)
Elected using plurality rule	0.212 [0.106, 0.358]	0.398 [0.272, 0.514]	0.311 [0.218, 0.405]	0.055 [0.026, 0.099]	0.020 [0.009, 0.038]
Elected using a runoff rule	0.152 [0.063, 0.293]	0.353 [0.243, 0.462]	0.378 [0.279, 0.465]	0.080 [0.038, 0.144]	0.031 [0.015, 0.057]
Difference (plurality→runoff)	-0.060 [-0.123, -0.004]	-0.045 [-0.118, 0.001]	0.066 [0.004, 0.131]	0.025 [0.001, 0.060]	0.010 [0.001, 0.025]
Percentage change	-28.3%	not significant	21.3%	46.2%	51.5%

95% confidence intervals are in brackets. Note that scores on the CIRI scale range from 0 to 8; higher values indicate greater government respect for human rights.

Starting with the ‘Best’ scenario in Table 7, you can see that the probability of an ‘8’ on the the *CIRI scale* (i.e. the highest possible score for government respect for human rights) is 0.509 when the president is elected using plurality rule, but is 0.614 when the president is elected using a runoff rule. Note that the difference in the probability going from 0.509 to 0.614 is 0.105, which—as you can see—is statistically significant at least at the the 95% level, given that the corresponding 95% confidence interval (which is in brackets) does not overlap with zero. Substantively, increasing a 0.509 probability by 0.105 is roughly a 21% increase. This means that in any given year, for a state that matches the ‘Best’ case parameters, that state is roughly 21% *more* likely to be at the highest level of government respect for human rights when the president had been elected using a runoff rule in the previous election.

Continuing with the ‘Best’ scenario, you can see that lower scores on the *CIRI scale* (i.e. scores of ‘4’ through ‘7’) all correspond with *negative* percent changes. At this point I would like to redirect the reader’s attention back to Table 6. Note that for the ‘Best’ scenario, a ‘8’ was used for the *previous year’s score on the CIRI scale* variable. Returning now to the ‘Best’ scenario portion of Table 7, note that any *CIRI scale* scores *less* than ‘8’ would indicate a *decrease* in government respect for human rights versus the previous year, which is indicated in the parentheses (e.g. a *CIRI scale* score of ‘7’ would be a 1-unit decrease in government respect for human rights versus the previous year, since the previous year for this scenario had a *CIRI scale* score of ‘8’ per Table 6). As such, the *negative* percent changes associated with scores ‘4’ through ‘7’ essentially indicate that for a state that matches the ‘Best’ case parameters, that state is *less* likely to experience a reduction in government respect for human rights (when compared to the previous year). Looking now to the ‘Average’ and ‘Worst’ scenarios, you can see that for both, relatively lower scores on the *CIRI scale* correspond with negative percent changes, while relatively higher scores on the *CIRI scale* correspond with positive percent changes.

## Conclusion

Davenport and Armstrong ([40]: 51) caution that there is no easy way to reduce repression, as the simple “adoption of some…elements will not automatically decrease repressive activity.” Rather, scholarship suggests that states should strive to slowly and steadily increase their national wealth, reduce overpopulation, and avoid civil conflict. On the contrary, this article suggest that perhaps it actually *is* possible to decrease repressive activity by simply adopting certain elements. While earlier research argues that *advancing* to a runoff round promotes human rights [41], in this article I have found that *the mere presence of a runoff rule* promotes human rights. This is good news for policy-makers as it suggests that simply adopting a runoff rule has the potential to improve human rights practices, even if subsequent elections do not advance to a runoff round.
